# Phylogenetic and ecological patterns in nighttime transpiration among five members of the genus *Rubus* co-occurring in western Oregon

**DOI:** 10.1002/ece3.1608

**Published:** 2015-08-06

**Authors:** Brandon McNellis, Ava R Howard

**Affiliations:** 1 Department of Biology, Western Oregon UniversityMonmouth, Oregon, 97361; 2 Department of Biology, University of California-RiversideRiverside, California, 92507

**Keywords:** Ecophysiology, gas exchange, nighttime transpiration, nocturnal transpiration, Rubus

## Abstract

Nighttime transpiration is a substantial portion of ecosystem water budgets, but few studies compare water use of closely related co-occurring species in a phylogenetic context. Nighttime transpiration can range up to 69% of daytime rates and vary between species, ecosystem, and functional type. We examined leaf-level daytime and nighttime gas exchange of five species of the genus *Rubus* co-occurring in the Pacific Northwest of western North America in a greenhouse common garden. Contrary to expectations, nighttime transpiration was not correlated to daytime water use. Nighttime transpiration showed pronounced phylogenetic signals, but the proportion of variation explained by different phylogenetic groupings varied across datasets. Leaf osmotic water potential, water potential at turgor loss point, stomatal size, and specific leaf area were correlated with phylogeny but did not readily explain variation in nighttime transpiration. Patterns in interspecific variation as well as a disconnect between rates of daytime and nighttime transpiration suggest that variation in nighttime water use may be at least partly driven by genetic factors independent of those that control daytime water use. Future work with co-occurring congeneric systems is needed to establish the generality of these results and may help determine the mechanism driving interspecific variation in nighttime water use.

## Introduction

During the night, stomates of C_3_ plants partially close to conserve water while there is no sunlight available to drive carbon fixation. The degree of stomatal closure, however, is variable between and within species and between plant functional groups and ecosystem types (Caird et al. 2007; Dawson et al. 2007). Rates of nighttime water loss commonly exceed 10% of daytime rates and can be as high as 69% (Caird et al. 2007; Dawson et al. 2007; Forster 2014). Numerous hypotheses have been put forth to explain the patterns of nighttime stomatal conductance (*g*_night_) and nighttime transpiration (*E*_night_*)* observed in natural systems, with significant consequences for plant growth and physiology as well as ecosystem water budgets and terrestrial ecosystem modeling (Caird et al. 2007; Domec et al. 2012; Zeppel et al. 2014). Most of these hypotheses focus on environmental drivers or patterns of occurrence in native communities or agronomic settings. Relatively little study has been made of the heritable variation in *g*_night_ among related taxa in a phylogenetic context.

Studies of *g*_night_ and *E*_night_ have revealed some consistent patterns in nature but as of yet no mechanism or consistent adaptive value has been definitively demonstrated (Caird et al. 2007; Forster 2014; Zeppel et al. 2014). *E*_night_ is downregulated in response to high vapor pressure deficit (Barbour and Buckley 2007; Ogle et al. 2012) and dry soil (Howard and Donovan 2007; Howard et al. 2009; Zeppel et al. 2011, 2012) and has been found to decrease the magnitude of hydraulically redistributed water by plant roots (Howard et al. 2009; Neumann et al. 2014). It has been suggested that nighttime canopy water loss may drive bulk flow of soil solution which could improve nutrient acquisition, aid in delivery of nutrients to distal plant organs, facilitate xylem embolism repair, deliver oxygen to parenchyma cells in woody tissue or increase carbon fixation in early morning. Support for adaptive ideas in the literature has been mixed (Daley and Phillips 2006; Marks and Lechowicz 2007; Scholz et al. 2007; de Dios et al. 2013) with some ideas receiving no support when investigated in controlled manipulative studies (Howard and Donovan 2007; Christman et al. 2009; Howard et al. 2009; Auchincloss et al. 2014). Efforts to tease apart support for the competing explanatory hypotheses would benefit from an improved understanding of how interspecific variation in *g*_night_ relates to evolutionary history.

Previous research examining *g*_night_ in closely related species has been relatively limited (Howard and Donovan 2007; Phillips et al. 2010). Some studies in systems of agricultural cultivars have found no differences between genotypes (Schoppach et al. 2014), while other work in model systems has found pronounced intraspecific variation (Christman et al. 2008). The effect that heritable variation in natural populations has on rates of nighttime water use is still poorly understood. The genus *Rubus* represents a convenient system to examine interspecific variation in *g*_night_ and *E*_night_ in co-occurring closely related species. The phylogeny of *Rubus* has been well studied using both morphological and molecular methods and is diverse enough in western Oregon that several separate subgenera can be represented in a single study (Hitchcock and Cronquist 1973; Alice and Campbell 1999). If all species co-occur, then observed differences in physiology may reflect adaptive niche differentiation (Ordonez et al. 2010).

In western Oregon, *Rubus armeniacus* (Himalayan blackberry) is listed as a noxious weed (Oregon Department of Agriculture, 2006). Both it and *R. laciniatus* (cutleaf blackberry) have become widespread since their introduction in the early 20th century (Clark et al. 2013). *Rubus spectabilis*, *R. parviflorus*, and *R. ursinus* are native species similarly widespread (Hitchcock and Cronquist 1973). Compared to native congeners, both *R. armeniacus* and *R. laciniatus* maintain higher photosynthetic rates (McDowell 2002) and *R. armeniacus* uses more water and produces many times more fruit and seed (McDowell and Turner 2002; Caplan and Yeakley 2010, 2013). Co-occurring native *Rubus* species often have similar ruderal habits and can all be found within the same community (Hitchcock and Cronquist 1973; Franklin and Dyrness 1988). While much is known about daytime physiology and water relations in several members of *Rubus* occurring in the Pacific Northwest (McDowell 2002; Caplan and Yeakley 2010), nighttime transpiration has not been measured. Rates of nighttime gas exchange are usually positively correlated to rates of daytime gas exchange (Caird et al. 2007) but have been shown to be decoupled in at least one study in a model system (Christman et al. 2008).

In this study, we sought to examine the patterns of nighttime gas exchange in a system of co-occurring *Rubus* species. We hypothesized that (1) variation in *g*_night_ and *E*_night_ is greater between than within species of *Rubus*; (2) a consistent phylogenetic signal is present in the patterns of interspecific variation in *g*_night_ and *E*_night_; (3) *g*_night_ and *E*_night_ are correlated to daytime gas exchange; and (4) other leaf-level physiological and anatomical parameters are partially responsible for observed variation in *g*_night_.

## Materials and Methods

### Sample collection

**Figure 1:**
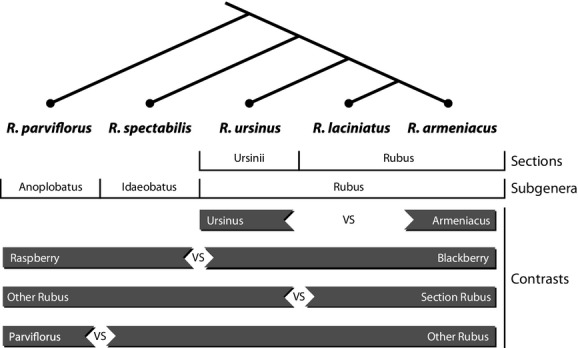
Phylogenetic relationship and preplanned contrasts for five *Rubus* species native to the Pacific Northwest of the United States. Phylogeny taken from Howarth et al. (1997) and Alice and Campbell (1999).

Five members of the genus *Rubus* were collected for study: *Rubus armeniacus* Focke*, Rubus laciniatus* (Weston) Willd., *Rubus parviflorus* Nutt.*, Rubus spectabilis* Pursh.*,* and *Rubus ursinus* Cham. & Schltdl. Three of the species (*R. parviflorus, R. spectabilis,* and *R. ursinus*) are native to the region in which they were collected (Hitchcock and Cronquist 1973), while two of the species (*R. armeniacus* and *R. laciniatus*) are invasive species native to Eurasia (Clark et al. 2013). A phylogeny was constructed (Fig.1) of the five species using morphological and genetic relationships published in the literature (Howarth et al. 1997; Alice and Campbell 1999). “Blackberries” include members of subgenus *Rubus* (section *Rubus*) that have fruits that do not separate from the receptacle at maturity. “Raspberries” include other members of genus *Rubus* (subgenus *Anoplobatus* and subgenus *Idaeobatus* in this study) that do not show this character. *Rubus ursinus* (subgenus *Rubus,* section *Ursinii*) was grouped here with the blackberries according to morphological characters, although more recent molecular work has identified it as a probable hybrid with uncertain placement. During phylogenetic contrast analyses, *R. parviflorus* was treated both as a raspberry grouped with *R. spectabilis (Idaeobatus)* on the basis of morphological characteristics (Alice and Campbell 1999) and as a representative of a subgenus basal relative to the others.

Eight individuals of each species were collected from four sites in the Suislaw National Forest, OR as well as four sites in the Willamette Valley, OR in September of 2010 and January of 2011. When possible, individuals of each species were collected at each site. If species were missing from particular sites, then additional individuals were taken from different locations within sites in which they were present. Plants used for sample collection within the same site were chosen to ensure that they were not physiologically continuous (not linked by canes or rhizomes). Individuals were immediately transported with intact root and stem systems back to Western Oregon University, Monmouth, OR. In the greenhouse, they were transplanted into 3.7 L plastic pots with Promax General Purpose Growing Medium (Premier Horticultural Inc, Quakertown, PA). Plants were watered daily.

### Experimental design

In January 2011, plants were arranged in a randomized complete block design in a heated greenhouse with 500 *μ*mol supplemental photosynthetically active radiation (PAR) provided by metal halide grow lights from 0700 to 1900 h. Plants were watered with drip irrigation or by hand twice a day to field capacity and fertilized with Miracle Grow Tomato Plant Food (18N/18P/21K, Miracle-Gro Products, Inc, Marysville, OH) approximately every 2 weeks. Additionally, applications of Osmocote Smart-Release Plant Food with micronutrients (16N/5P/10K, Scotts-Sierra Horticultural Products Company, Marysville, OH) were given in January of 2011, April 2011, and April 2012. Plants canopies were regularly cut back, and roots were pruned in May of 2012 with new soil being added to all pots postroot pruning. *Rubus laciniatus* was removed from the experimental design in August 2012 due to pest susceptibility. Plant health and instrument failure resulted in omitting some plant replicates from datasets resulting in unbalanced designs – replicates are noted within each section of measurement procedures.

### Gas exchange

Daytime gas exchange was measured three times over the course of the study on 8 April 2011, 29 July 2011, and 3–4 June 2013 using a LI-6400 Portable Photosynthesis System (LiCor Inc., Lincoln, NE). Seven individuals of *R. ursinus* and eight individuals of all other species were used for the 2011 datasets, but one individual of *R. parviflorus* and three individuals of *R. ursinus* were removed before the 2013 dataset. Daytime chamber light was supplied at 1500 to 1800 *μ*mol m^−2^ sec^−1^ PAR by the LI-6400 02B red/blue light source (LiCor Inc.). Daytime chamber CO_2_ concentration ranged from 344 to 391 ppm. Chamber water mole fraction was controlled to maintain relative humidity within the chamber at 5–15% above ambient conditions to compensate for the absence of a leaf boundary layer within the measurement chamber. Leaf temperature was controlled at a temperature that approximated average greenhouse temperature on the day of measurement. Daytime measures were taken between 0900 and 1500 h. For all datasets, the youngest fully expanded sun-exposed leaf was chosen for measurements. Plants were watered at least 1 h prior to measurement to ensure water stress did not affect measurement of gas exchange rates. Instantaneous water-use efficiency (WUE) was calculated by taking leaf-level measurements of maximum photosynthesis and dividing by concurrent measures of transpiration.

Gas exchange at night was measured starting in the late evening of 8 April 2011, 25 July 2011, and 2–3 June 2013 using a LI-6400 with replication identical to the daytime datasets. Chamber humidity ranged from 42 to 62%, and control of flow rate and chamber water mole fraction was used to target a chamber humidity that was approximately 5–15% above ambient. Measurements were taken between astronomical sunset and astronomical sunrise (between 2300 and 0430 h). Gas exchange at night was measured on the same leaf as daytime gas exchange. Rarely, leaves used for daytime measurement were damaged and an adjacent leaf was used for measurement. Nighttime measurements were performed in darkness with the aid of headlamps filtered with plastic that transmitted only monochromatic green light at an intensity undetectable by a PAR meter (LI-190, LiCor Biosciences Inc., Lincoln, NE). Plants were watered at least 1 h prior to gas exchange measurements.

Minimum leaf transpiration was assessed in July 2011 on four individuals of each species randomly selected from the eight present in the experimental design. Measures were taken by weighing individual cut leaves as they dried down on the laboratory bench along with simultaneous measurement with a LI-6400 during the period of constant rate water loss (Howard and Donovan 2007). Minimum leaf transpiration estimated by weighing and by the LI-6400 was significantly correlated (*r*^2^ = 0.79, *P* < 0.0001, *n* = 20), and only LI-6400 measures are further analyzed and presented here.

### Leaf water potential and hydraulic resistance measurements

Leaf water potentials were taken using a PMS Model 1000 Pressure Bomb (PMS Instrument Company, Albany, OR) immediately after gas exchange sets in the mid-afternoon (mid-day, Ψ_md_) and very early morning (predawn, Ψ_pd_) in July 2011 and June 2013 using unbagged freely transpiring leaves. Measurements were made on the same leaf used for gas exchange or a leaf adjacent to the gas exchange leaf. In 2011, leaves were sampled from each of eight *R. armeniacus* and *R. parviflorus* individuals, seven *R. laciniatus* and *R. spectabilis* individuals, and six *R. ursinus* individuals. In 2013, two additional leaves were covered with aluminum foil and placed in plastic bags near the base of each plant. One of these bagged leaves was measured at mid-day and was expected to equilibrate with the water potential of the xylem within the stem immediately adjacent to the leaf (Ψ_xylem_), while the other was measured predawn and was expected to equilibrate with the soil (Ψ_maximum_). In 2013, transpiring and nontranspiring leaves were sampled from each of eight *R. armeniacus* individuals, seven *R. parviflorus* and *R. spectabilis* individuals, and four *R. ursinus* individuals.

Root (*R*_root_), shoot (*R*_shoot_), whole-plant daytime (*R*_plant_), and whole-plant nighttime (*R*_pdplant_) hydraulic resistances were calculated from 2013 water potential and gas exchange data using the following formulas (modified from Nardini et al. 2003):

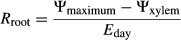
1

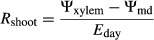
2

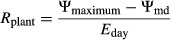
3

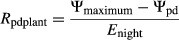
4

### Leaf anatomical and physiological traits

Specific leaf area (SLA) was measured in November 2011 on five leaves taken from four individuals of each species. Leaf area was measured on hydrated leaves using a flatbed scanner and image analysis software (ImageJ; National Institute of Health, Bethesda, Maryland, USA). Dry weight was measured by drying leaves at 60°C in a drying oven for at least 48 h. Adaxial and abaxial leaf surface impressions were taken on five fully hydrated leaves of six *R. armeniacus*, five *R. parviflorus*, four *R. spectabilis*, and three *R. ursinus* individuals, respectively, in June 2013 using vinyl polysiloxane. Adaxial leaf surfaces showed no stomata, and all values reported here are for the abaxial surface. Slides of the molds were created using a thin layer of toluene sulfonamide/formaldehyde resin and were photographed using a microscope camera. Stomatal size, length, width, and density were measured by analyzing the slide photographs using ImageJ. Length and width were calculated from an ellipse fitted to each stomata. Size, length, and width measurements included both guard cells and stomatal pore.

Pressure–volume curves were constructed on four leaves of four individuals each of *R. armeniacus*, *R. spectabilis*, and *R. ursinus* in March and April 2013 using the bench dry method (Tyree and Hammel 1972). This process was continued for each leaf until enough data were obtained to generate a curve describing the relationship between leaf water content and water potential (Koide et al. 2000). Leaf osmotic potential (Ψ_o_), leaf water potential at turgor loss point (Ψ_TLP_), leaf bulk modulus of elasticity, and leaf capacitance were calculated from the curves and averaged (Koide et al. 2000).

### Statistical analysis

Data were analyzed in SAS (version 9.1; SAS Institute Inc. Cary, NC) using a mixed-model ANOVA (PROC MIXED) with block as random effect and species as fixed effect or with a general linear model (PROC GLM) when a subset of replicates were measured. Data were log and square root transformed where necessary to approach model assumptions for normality of residuals and homogeneity of variance. Independent preplanned contrasts (Fig.1) to test phylogenetic and ecological signals within the data were included in the ANOVAs. Effect sizes (*r*^2^_effect size_), which measure the proportion of total variation that is explained by a contrast, were calculated in MS Excel from model outputs according to formulae given by Furr (2004). Pearson correlations and associated significance levels were assessed with PROC CORR in SAS.

## Results

### Gas exchange

**Figure 2:**
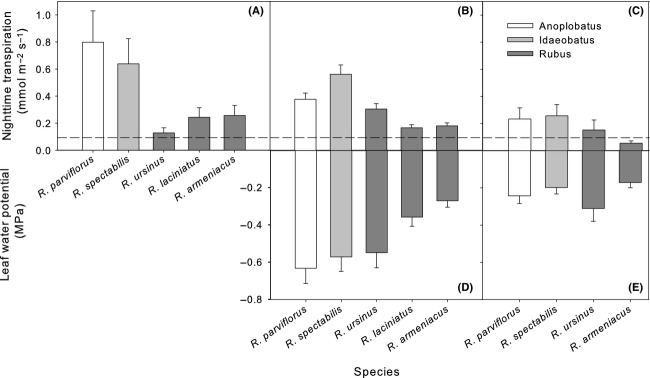
Nighttime transpiration and predawn leaf water potential of five *Rubus* species, three blackberry (subgenus *Rubus*) and two raspberry (subgenus *Idaeobatus* and subgenus *Anoplobatus*). Measures were taken during April 2011 (A), July 2011 (B, D), and June 2013 (C, E). Bars are ls means (+SE). The dashed line represents the average minimum leaf transpiration rate measured in July 2011 and found to be the same for all species.

**Figure 3:**
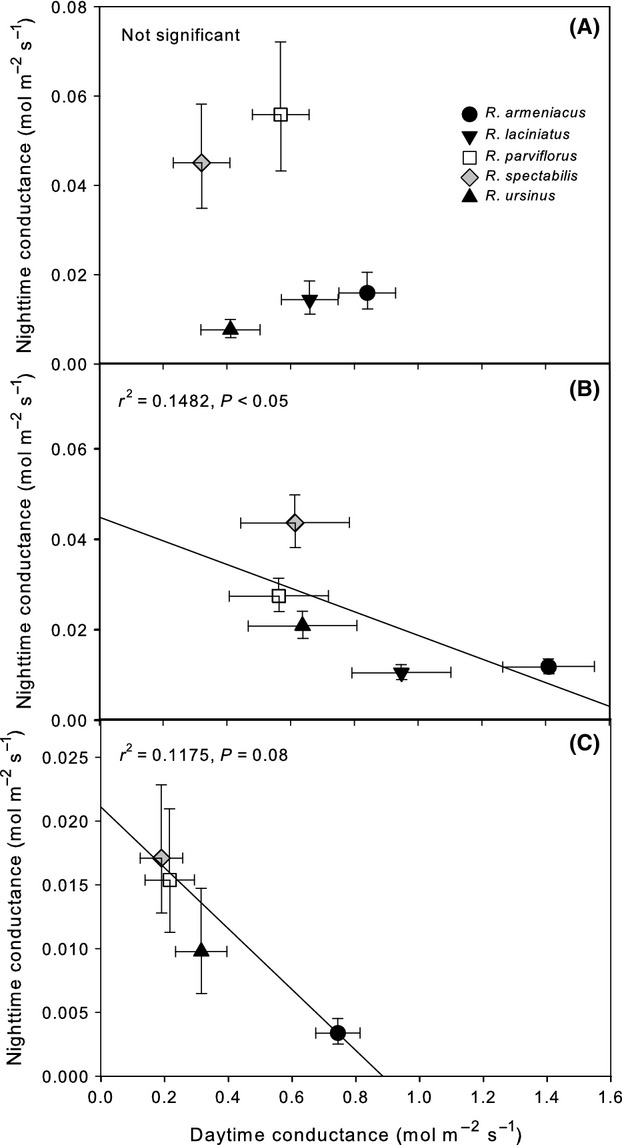
Nighttime versus daytime leaf conductance in five *Rubus* species, three blackberry (subgenus *Rubus*) and two raspberry (subgenus *Idaeobatus* and subgenus *Anoplobatus*). Measures were taken during April 2011 (A), July 2011 (B), and June 2013 (C). Points are ls means (±SE).

Nighttime conductance and transpiration were measured above instrument error for all species. Rates of gas exchange ranged across the genus from 0.0034 to 0.0558 mol m^−2^ sec^−1^ for *g*_night_ and from 0.0531 to 0.7980 mmol m^−2^ sec^−1^ for *E*_night_ (Table S1) and represent from less than 1% of daytime rates to 14% of daytime rates. The lowest rates of *g*_night_ and *E*_night_ were for *R. armeniacus* in June 2013 and the highest for *R. parviflorus* and *R. spectabilis* in April 2011 (Figs.2, 3). Minimum leaf conductance and transpiration measured on excised leaves did not differ between species (*P* > 0.3) and was generally lower than measures of *g*_night_ and *E*_night_ on intact leaves in the greenhouse (Fig.2). Averaged across species, minimum leaf conductance was 0.0054 (±0.0006) mol m^−2^ sec^−1^ and minimum leaf transpiration was 0.094 (±0.011) mmol m^−2^ sec^−1^.

**Figure 4:**
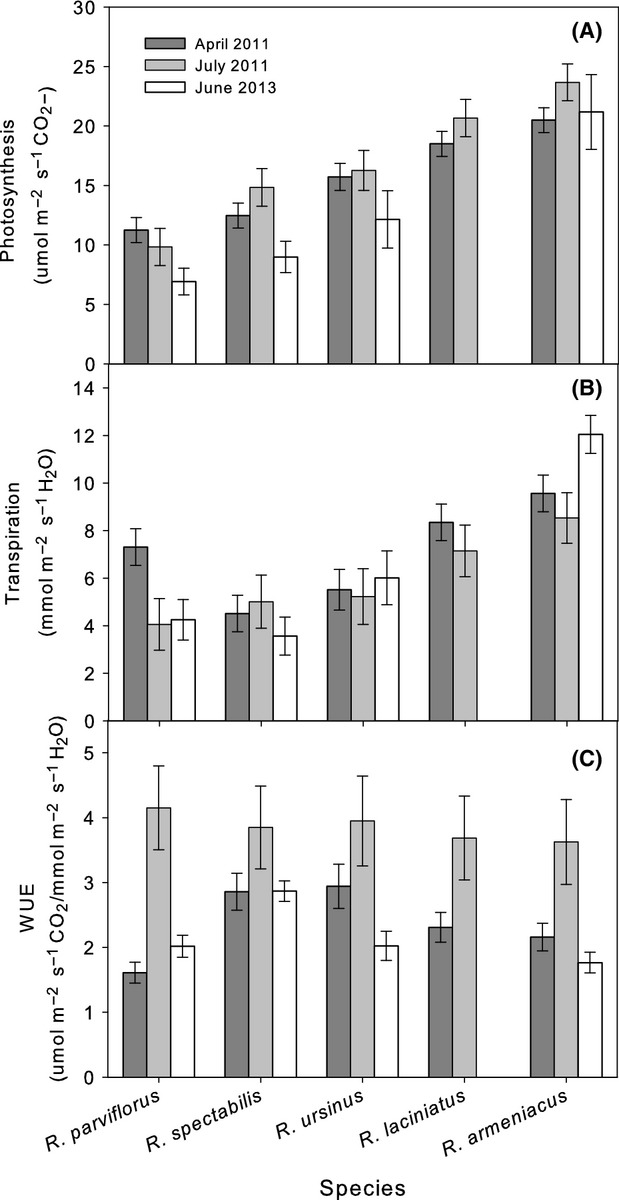
Concurrently measured photosynthesis (A), transpiration (B), and water-use efficiency (WUE; C) for five *Rubus* species, three blackberry (subgenus *Rubus*) and two raspberry (subgenus *Idaeobatus* and subgenus *Anoplobatus*). Bars are ls means (+SE).

On all three sampling dates, species differed significantly for *g*_night_, *E*_night_, *g*_day_, *E*_day,_ and photosynthesis. Evidence of a significant interspecific signal connected to phylogeny was evident in all gas exchange measures (Table1). The raspberry versus blackberry and section *Rubus* versus other *Rubus* contrasts (Fig.1) tended to explain more of the interspecific variation in water-use measures than did the *R. armeniacus* versus *R. ursinus* and *R. parviflorus* versus other *Rubus* contrasts although there were sometimes large differences in effect size between sampling dates (Table1). Nighttime conductance and transpiration showed a strong phylogenetic signal. In April 2011, the raspberry versus blackberry contrast explained 66% of all variation (94% of explained variation) in *g*_night_ and 65% of all variation (92% of explained variation) in *E*_night_ (Table1). This effect was reduced but still substantial in July 2011 when this contrast explained 53% of variation in *g*_night_ and 51% of variation in *E*_night_ as well as in June 2013 where it explained 24% of all variation in both *g*_night_ and *E*_night_. The section *Rubus* versus other *Rubus* contrast was the second most successful in explaining variation in *g*_night_ and *E*_night_ and was largest in July 2011. Daytime parameters, including A, *g*_day,_ and *E*_day,_ were best explained by the section *Rubus* versus other *Rubus* contrast (Table1). Water-use efficiency differed significantly between species only in April 2011 and June 2013 (Fig.4, Table S1). In these datasets, the *R. parviflorus* versus other *Rubus* contrast explained the most variation in April 2011 and the raspberry versus blackberry contrast explained the most variation in June 2013 (Table1).

**Table 1:** Effect size and significance levels for independent contrasts in ANOVAs on gas exchange traits measured on four to five *Rubus* species

			Leaf-level gas exchange					
Contrast			Day A	Day g	Day E	Night g	Night E	WUE
*R. arm*. – *R. urs*.	2011 April	*r*^2^	0.1120	0.2590	0.2211	0.0648	0.0720	0.0805
	Significance	***F***_**1,32**_** = 12.05** ***P***** < 0.01**	***F***_**1,32**_** = 17.82** ***P***** < 0.001**	***F***_**1,32**_** = 14.46** ***P***** < 0.001**	***F***_**1,32**_** = 7.11** ***P***** < 0.05**	***F***_**1,32**_** = 7.91** ***P***** < 0.01**	***F***_**1,32**_** = 4.59** ***P***** < 0.05**	
	2011 July	*r*^2^	0.1760	0.2631	0.1703	0.0825	0.0928	–
	Significance	***F***_**1,23**_** = 19.42** ***P***** < 0.001**	***F***_**1,23**_** = 11.86** ***P***** < 0.01**	***F***_**1,23**_** = 7.01*****P***** < 0.05**	***F***_**1,31**_** = 8.71** ***P***** < 0.01**	***F***_**1,31**_** = 9.56** ***P***** < 0.01**		
	2013 June	*r*^2^	0.1042	0.2452	0.2095	0.1084	0.1112	0.0183
	Significance	***F***_**1,22**_** = 6.89** ***P***** < 0.05**	***F***_**1,22**_** = 24.96** ***P***** < 0.0001**	***F***_**1,22**_**=19.02** ***P***** < 0.001**	***F***_**1,22**_** = 4.47** ***P***** < 0.05**	***F***_**1,22**_** = 4.59** ***P***** < 0.05**	*F*_1,22_ = 0.90 NS	
Rasp. – black.	2011 April	*r*^2^	0.5378	0.1406	0.1298	0.6628	0.6518	0.0391
	Significance	***F***_**1,32**_** = 57.87** ***P***** < 0.0001**	***F***_**1,32**_** = 9.67** ***P***** < 0.01**	***F***_**1,32**_** = 8.49** ***P***** < 0.01**	***F***_**1,32**_** = 72.75** ***P***** < 0.0001**	***F***_**1,32**_** = 71.49** ***P***** < 0.0001**	*F*_1,32_ = 2.23 NS	
	2011 July	*r*^2^	0.4556	0.1713	0.2131	0.5259	0.5088	–
		Significance	***F***_**1,23**_** = 50.28** ***P***** < 0.0001**	***F***_**1,23**_** = 7.72** ***P***** < 0.05**	***F***_**1,23**_** = 8.77** ***P***** < 0.01**	***F***_**1,31**_** = 55.53** ***P***** < 0.0001**	***F***_**1,31**_** = 52.43** ***P***** < 0.0001**		
	2013 June	*r*^2^	0.3962	0.3312	0.3522	0.2416	0.2385	0.1898
	Significance	***F***_**1,22**_** = 26.20** ***P***** < 0.0001**	***F***_**1,22**_** = 33.72** ***P***** < 0.0001**	***F***_**1,22**_** = 31.98** ***P***** < 0.0001**	***F***_**1,22**_** = 9.96** ***P***** < 0.01**	***F***_**1,22**_** = 9.84** ***P***** < 0.01**	***F***_**1,22**_** = 9.33** ***P***** < 0.01**	
Sect. *Rubus* – others	2011 April	*r*^2^	0.5328	0.3795	0.3647	0.1067	0.0917	0.0096
	Significance	***F***_**1,32**_** = 57.33** ***P***** < 0.0001**	***F***_**1,32**_** = 26.11** ***P***** < 0.0001**	***F***_**1,32**_** = 23.85** ***P***** < 0.0001**	***F***_**1,32**_** = 11.71** ***P***** < 0.01**	***F***_**1,32**_** = 10.06** ***P***** < 0.01**	*F*_1,32_ = 0.55 NS	
	2011 July	*r*^2^	0.5680	0.3549	0.3598	0.5443	0.5485	–
	Significance	***F***_**1,23**_** = 62.68** ***P***** < 0.0001**	***F***_**1,23**_** = 16.00** ***P***** < 0.001**	***F***_**1,23**_** = 14.81** ***P***** < 0.001**	***F***_**1,31**_** = 57.48** ***P***** < 0.0001**	***F***_**1,31**_** = 56.52** ***P***** < 0.0001**		
	2013 June	*r*^2^	0.4942	0.7004	0.6539	0.3871	0.3897	0.1613
	Significance	***F***_**1,22**_** = 32.68** ***P***** < 0.0001**	***F***_**1,22**_** = 71.30** ***P***** < 0.0001**	***F***_**1,22**_** = 59.37** ***P***** < 0.0001**	***F***_**1,22**_** = 15.96** ***P***** < 0.001**	***F***_**1,22**_** = 16.08** ***P***** < 0.001**	***F***_**1,22**_** = 7.93** ***P***** < 0.05**	
*R. *parv. – others	2011 April	*r*^2^	0.2788	0.0003	0.0026	0.3238	0.3306	0.3226
	Significance	***F***_**1,32**_** = 30.00** ***P***** < 0.0001**	*F*_1,32_ = 0.02 NS	*F*_1,32_ = 0.17 NS	***F***_**1,32**_** = 35.54** ***P***** < 0.0001**	***F***_**1,32**_** = 36.26** ***P***** < 0.01**	***F***_**1,32**_** = 18.39** ***P***** < 0.001**	
	2011 July	*r*^2^	0.4245	0.0825	0.1492	0.0693	0.0661	–
	Significance	***F***_**1,23**_** = 46.85** ***P***** < 0.0001**	*F*_1,23_ = 3.72 NS	***F***_**1,23**_** = 6.14** ***P***** < 0.05**	***F***_**1,31**_** = 7.32** ***P***** < 0.05**	***F***_**1,31**_** = 6.81** ***P***** < 0.05**		
	2013 June	*r*^2^	0.2655	0.0988	0.0951	0.0701	0.0703	0.0208
	Significance	***F***_**1,22**_** = 17.56** ***P***** < 0.001**	***F***_**1,22**_** = 10.06** ***P***** < 0.01**	***F***_**1,22**_** = 8.63** ***P***** < 0.01**	*F*_1,22_ = 2.89 NS	*F*_1,22_ = 2.90 NS	*F*_1,22_ = 1.02 NS	

“NS” denotes a not significant contrast. Note, in 2013, *R. laciniatus* was not included in the study which slightly alters all but the *R. armeniacus* vs *R. ursinus* (arm-urs) contrast. In July 2011, the main effect of “species” in the ANOVA for WUE was not significant. Bold values indicate statistically significant contrasts at α = 0.05.

Subgenus *Rubus* (blackberries), which groups *R. ursinus* with section *Rubus*, had 25–40% lower *g*_night_ and 29–46% lower *E*_night_ than members of the other subgenera (*Idaeobatus* and *Anoplobatus*, raspberries), and this relationship was significant in all datasets (*P* < 0.01, Table1, Fig.2). Mean *g*_night_ and *E*_night_ as a percentage of *g*_day_ and *E*_day_ was 2–3% for the blackberries and 9–10% for the raspberries. *Rubus parviflorus* (subgenus *Anoplobatus*) grouped with *R. spectabilis* (subgenus *Idaeobatus*) for all gas exchange measurements (*P* > 0.05) while *R. armeniacus* and *R. laciniatus* (subgenus *Rubus* sect. *Rubus*) grouped together for all nighttime datasets where both were included and differed during the day only for *g*_day_ in April 2011 (Fig.3). *Rubus ursinus* (subgenus *Rubus* sect. *Ursinii*) did not show a clear pattern across datasets and grouped with subgenus *Rubus* in April 2011, subgenus *Idaeobatus* in July 2011 and was not significantly different from any other species in June 2013. Water-use efficiency had variable patterns of effect size among the phylogenetic preplanned contrasts in the April 2011 and June 2013 datasets where there was a significant main effect of species (Fig.4). In general, *R. spectabilis* trended higher than *R. parviflorus* among the raspberries and *R. ursinus* trended slightly higher than section *Rubus*.

### Water potentials and hydraulic resistance

Measures of Ψ_pd_ and Ψ_md_ were overall more negative in July 2011 compared to June 2013. Significant differences between species were found for Ψ_pd_ in July 2011 and Ψ_maximum_ in June 2013 (Table S1). In July 2011, Ψ_pd_ was an average 0.25 MPa less negative in section *Rubus* (Table2; Fig.2). In June 2013, Ψ_maximum_ of subgenus *Rubus* (*R. armeniacus and R. ursinus*) was an average 0.08 MPa less negative than the raspberries. All other measures of plant water potential were not significantly different between species.

**Table 2:** Significance levels for preplanned contrasts in ANOVAs for those physiological and anatomical traits for which the main effects of species were significant

Contrast		Traits						
		Leaf water potential	Pressure–volume curve^1^		Stomatal anatomy^2^	Hydraulic resistance^2^		
		Ψ_PD_ 2011	Ψ_O_	Ψ_TLP_	Stoma size	*R*_plant_	*R*_root_	*R*_plantPD_
Arm-urs	*r*^2^	0.1975	0.5188	0.5794	0.0098	0.0891	0.0601	0.0120
	Significance	***F***_**1,31**_** = 11.33** ***P***** < 0.01**	***F***_**1,9**_** = 10.19** ***P***** < 0.05**	***F***_**1,9**_** = 12.55** ***P***** < 0.01**	*F*_1,14_ = 0.98 NS	*F*_1,20_ = 3.86 NS	*F*_1,20_ = 2.27 NS	*F*_1,20_ = 0.46 NS
Rasp. – black	*r*^2^	0.2190	0.0229	0.0055	0.0634	0.3738	0.3302	0.4316
	Significance	***F***_**1,31**_** = 12.56** ***P***** < 0.01**	*F*_1,9_ = 0.45 NS	*F*_1,9_ = 0.12 NS	***F***_**1,14**_** = 6.32** ***P***** < 0.05**	***F***_**1,20**_** = 16.20** ***P***** < 0.001**	***F***_**1,20**_** = 12.48** ***P***** < 0.01**	***F***_**1,20**_** = 16.53** ***P***** < 0.001**
Sect. *Rubus* – others	*r*^2^	0.3928	0.3004	0.3864	0.0652	0.3957	0.3128	0.2352
	Significance	***F***_**1,31**_** = 22.53** ***P***** < 0.0001**	***F***_**1,9**_** = 5.90** ***P***** < 0.05**	***F***_**1,9**_** = 8.37** ***P***** < 0.05**	***F***_**1,14**_** = 6.50** ***P***** < 0.05**	***F***_**1,20**_** = 17.15** ***P***** < 0.001**	***F***_**1,20**_** = 11.82** ***P***** < 0.01**	***F***_**1,20**_** = 9.01** ***P***** < 0.01**
Parv – other Rubus	*r*^2^	0.1227	–	**–**	0.7110	0.0976	0.0508	0.1755
	Significance	***F***_**1,31**_** = 7.04** ***P***** < 0.05**			***F***_**1,14**_** = 70.85** ***P***** < 0.0001**	*F*_1,20_ = 4.23 *P* = 0.05	*F*_1,20_ = 1.92 NS	***F***_**1,20**_** = 6.72** ***P***** < 0.05**

Measures were made on three to five *Rubus* species. Note cases where only a subset of the five species were measured. Bold values indicate statistically significant contrasts at α = 0.05.

1 *Rubus laciniatus* and *Rubus parviflorous* were absent.

2 *Rubus laciniatus* was absent.

**Figure 5:**
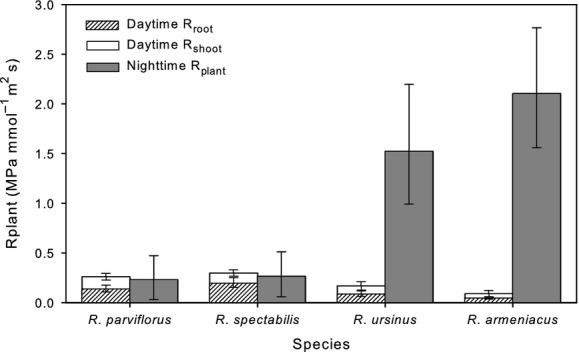
Midday and predawn hydraulic resistance (*R*_plant_) in four *Rubus* species, two blackberry (subgenus *Rubus*) and two raspberry (subgenus *Idaeobatus* and subgenus *Anoplobatus*). Midday measures are partitioned into root resistance (*R*_root_) and shoot resistance (*R*_shoot_). Measures were taken during June 2013. Bars are ls means (±SE).

Hydraulic resistance was significantly different between species for measures of *R*_root_, *R*_plant,_ and *R*_pdplant_, but not for *R*_shoot_ (Fig.5; Table S1). Daytime *R*_plant_ was significantly related to phylogenetic relationship (Table2). Blackberries had lower *R*_plant_ than raspberries, with *R. armeniacus* exhibiting the lowest *R*_plant_ of the four species measured. *R*_root_ followed this same pattern of interspecific variation. At night, a strong and directionally opposite phylogenetic pattern existed with blackberries exhibiting much higher *R*_pdplant_ than the raspberries, and with *R. armeniacus* exhibiting the highest *R*_pdplant_ of the four species measured (Table2; Fig.5).

### Leaf anatomical and physiological traits

**Figure 6:**
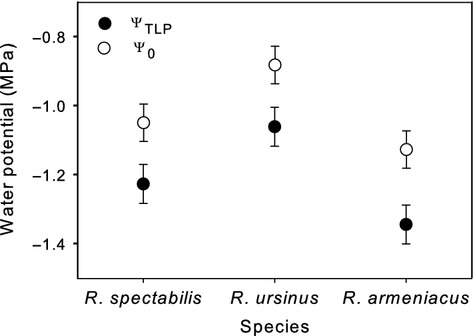
Leaf osmotic potential at full hydration (Ψ_0_) and leaf water potential at turgor loss point (Ψ_TLP_) derived from pressure–volume curves in three *Rubus* species. Points are ls means (±SE).

**Figure 7:**
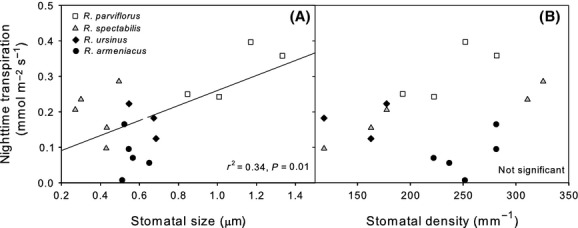
Nighttime transpiration measured in June 2013 versus stomatal size (A) and abaxial stomatal density (B) in four *Rubus* species. Points are individual plant replicates.

Other leaf-level anatomical and physiological traits were variously associated with phylogeny (Table2). Leaf osmotic potential (Ψ_o_) and water potential at turgor loss point (Ψ_TLP_) were significantly related to the section *Rubus* versus other *Rubus* contrast. *Rubus armeniacus* had significantly more negative Ψ_o_ and Ψ_TLP_ than *R. ursinus* with *R. spectabilis* not significantly different from either (Fig.6). Area-normalized leaf capacitance and stomatal density were not significantly different between species (*P* > 0.05; Table S1), although stomatal density in *R. ursinus* trended 33% lower than the combined mean of other species (Fig.7). Stomatal size was similar for all species except for *R. parviflorus,* which had stomata twice as large as the other species (Fig.7). This pattern appeared to be driven by both greater stomatal length (30%) and width (73%) in *R. parviflorus* compared to the average of all other species (Fig.7). Specific leaf area showed a phylogenetic relationship that appeared to largely be driven by the highly dissected leaves of *R. laciniatus*, which was the only significantly different species and had 33% lower SLA than the average of all others (Table S1).

## Discussion

All five *Rubus* species showed substantial and divergent nighttime water use during all measurement times, supporting our first hypothesis (Table1, Fig.2). Daytime measures of photosynthesis, *g*_day_ and *E*_day_ largely agreed with previous research on our study species (McDowell 2002; Caplan and Yeakley 2010). Rates of nighttime transpiration documented across a wide phylogenetic and ecological array of species, including closely related confamilial species in the genus *Rosa,* are generally in the range of 5–15% of *E*_day_ (Caird et al. 2007). This corresponds well with the range we found for *R. spectabilis* and *R. parviflorus*. Curiously, *R. armeniacus* and *R. laciniatus* did not show this pattern and instead maintained very low rates of nighttime water use relative to daytime. *Rubus ursinus*, a putative hybrid between subgenus *Rubus* and subgenus *Idaeobatus*, showed generally low rates of nighttime water use relative to daytime (Figs.2, 3) but could not be consistently grouped with either the raspberries or the blackberries in subgenus *Rubus* section *Rubus*.

Our second hypothesis was supported by the presence of a pronounced phylogenetic signal in all datasets. The proportion of variation in gas exchange explained by this signal was substantial, but its pattern was not consistent (Table1). The blackberry members of subgenus *Rubus* displayed lower *g*_night_ and *E*_night_ than the co-occurring raspberry species (Fig.2) reflecting shared genetic background. *Rubus spectabilis* and *R. parviflorus* did not display divergent patterns of gas exchange despite being more distantly related to each other than *R. armeniacus* and *R. laciniatus* are to *R. ursinus* (Alice and Campbell 1999; Clark et al. 2013). This relationship was also evident in preplanned contrasts, where the *R. parviflorus* versus other *Rubus* and *R. armeniacus* versus *R. ursinus* contrasts tended to explain less variation in the data relative to the raspberry versus blackberry contrast (Table1).

Phylogenetic effects on *g*_night_ and *E*_night_ were most evident among the *Rubus* species in April 2011, after which its influence decreased (Table1). This decline followed an overall decline in mean *g*_night_ and *E*_night_ across all species and appears to be driven by a decrease in nighttime water use by *R. parviflorus* and *R. spectabilis* (Fig.2). These decreases may be a response to mild water stress from increased root binding in pots or a response to slightly higher vapor pressure deficit in the greenhouse at night in the June and July months. These data allow confirmation of our second hypothesis but prevent us from anything beyond speculation on factors driving phylogenetic differences.

No obvious mechanism exists that explains nighttime transpiration in plants (Zeppel et al. 2014). However, a Mediterranean climate is common across much of the range of the study species, and soil moisture has been connected to rates of nighttime water use (Howard and Donovan 2007), suggesting that *g*_night_ may be influenced by adaptive pressure associated with drought. All of the blackberries had very conservative patterns of nighttime water use with *R. armeniacus* and *R. laciniatus* displaying rates less than 3% of daytime rates and *R. ursinus* less than 6%. Caplan and Yeakley (2013) found that daytime water-use physiology in *R. armeniacus* and *R. ursinus* showed a similar response to drought, contrasting with *R. parviflorus* and *R. spectabilis*. Additionally, numerous water conservation adaptations have been associated with *R. armeniacus* (McDowell 2002; Caplan and Yeakley 2013). The trend toward lower *g*_night_ and *E*_night_ in the blackberries may reflect the tendency of subgenus *Rubus* to invade microenvironments more prone to water stress than the raspberries in our study (Caplan and Yeakley 2006) and suggests that water-limited environments could provide some measure of ecological pressure on selection against high rates of nighttime water use. Results for Ψ_TLP_ and Ψ_o_ were partly consistent with these microhabitat specializations (Fig.6). The broad connections between the natural history of our study species and observed trends in *g*_night_ suggest that finer examination of patterns of nighttime water use in this system may prove useful in disentangling putative adaptive mechanisms from one another.

Contrary to expectations, nighttime gas exchange was either not correlated or weakly negatively correlated with daytime gas exchange (Fig.3). This finding opposes a significant body of previous research and refutes our third hypothesis. Most studies show that species with high daytime rates of water use tend to have relatively high rates of nighttime water use (Snyder et al. 2003; Christman et al. 2008; Ogle et al. 2012). The disconnect observed between daytime and nighttime water use warrants particular consideration as evidence against selection on nighttime transpiration as a neutral or daytime-correlated trait, at least in this system. Some evidence exists that there may be genetic controls for nighttime transpiration that disconnect it from daytime water use. Christman et al. (2008) found that crossing two natural accessions of *Arabidopsis* with genetic material from accessions that had divergent water-use physiology generated plants that displayed different *g*_day_ (or *g*_night_) from their parents while maintaining similar *g*_night_ (or *g*_day_). This suggests that *g*_day_ and *g*_night_ may be under separate genetic control despite often being highly correlated. While we cannot definitively connect any particular adaptive value or pressure with divergent selection on day and nighttime gas exchange characteristics, further study should be made to determine whether this occurs in other systems of co-occurring species and in what ecological context it can arise.

Other measures of leaf anatomical and physiological traits did not appear to adequately explain interspecific differences in *g*_night_ and *E*_night_, and we remain unable to support or reject our fourth hypothesis. Nighttime transpiration was not consistently correlated to photosynthetic rate (Figs.3, 4). This is in agreement with recent findings which suggest that predawn stomatal opening is not an adaptation for increased carbon gain (Auchincloss et al. 2014). Minimum leaf conductance was generally much less than *g*_night_ and did not differ significantly between species (Table S1). Therefore, significant interspecific variation between the *Rubus* species in this common garden appeared to be due to genetically based differences in stomatal regulation at night as opposed to anatomical limitations to shutting stoma or limiting cuticular water loss.

Soil moisture availability can potentially drive differences in nighttime water use (Howard and Donovan 2007; Zeppel et al. 2012) and was assessed during our 2013 gas exchange measurements as Ψ_maximum_. However, the correlation observed in our data was for approximately 0.1 MPa more negative soil water potentials associated with the higher transpiring raspberry species (Fig.2, Table S1). This suggests that differences in water potentials were not driven by interspecific variation in *E*_night_ – if water status was limiting transpiration, then we would expect the blackberries to have the more negative soil water potentials. There was a trend for *R. ursinus* to have lower stomatal density than other congenerics (Fig.7), but this pattern does not adequately explain observed differences in water use. *Rubus parviflorus* had significantly larger stomata than other species, potentially influencing its high rates of nighttime water use; however, *R. spectabilis* did not differ in stomatal size from any other species besides *R. parviflorus* (Fig.7).

While some measures of leaf anatomy and physiology were inadequate to explain patterns of interspecific variation in *g*_night_, multiple other factors could have influence. For example, two of our three blackberry species (*R. armeniacus* and *R. laciniatus*, subgenus *Rubus* section *Rubus*) are non-native to western Oregon (Clark et al. 2013). Invasive *Rubus* species transpired significantly less at night compared to native congenerics across all datasets (Table1, Fig.2) reflecting a conservative strategy relative to co-occurring natives. Conservative *E*_night_ may be an adaptive character of *Rubus* subgenus *Rubus* that aids in establishment and competitive dominance of alien species. Low *g*_night_ and *E*_night_ may represent a resource use efficiency advantage (Funk and Vitousek 2007). Other traits associated with accessing and storing water during drought, such as large root systems and wide, low-density canes, have already been associated with *R. armeniacus* (Caplan and Yeakley 2013). Daytime WUE in our study was not strongly associated with ecological status as an invasive or native (Table1; Fig.4) which may be due to measuring plants during morning hours and under conditions of high water availability. However, the only native species (*R. ursinus*) within subgenus *Rubus* also displayed intermediate to low rates of *g*_night_ and *E*_night_ (Fig.2). The strong presence of invasive character in the *R. fruticosus* aggregate suggests that ecology may be difficult to separate from evolutionary history in this group. Nevertheless, future studies incorporating the effect of low resource conditions on *g*_night_ and *E*_night_ in invasive plants may help integrate nighttime water use into existing theoretical frameworks describing resource use in invaded systems.

Polyploidy has long been noted as a potential factor influencing plant ecology and evolution (Ramsey and Ramsey 2014; Soltis et al. 2014), and *Rubus* species vary greatly in ploidy level. Polyploids have been found to be both more and less tolerant of drought stress than diploids (Li et al. 1996; Buggs and Pannell 2007; Mráz et al. 2014). Changes in anatomy resulting from increased cell size could potentially increase gas exchange and drought resistance (Li et al. 1996; Maherali et al. 2009) although variation in rates of nighttime water use in this study was not well explained by measured anatomical or physiological traits. *Rubus parviflorus* and *R. spectabilis* are predominantly diploid (Thompson 1997). *Rubus ursinus* is a 6×–12× polyploid complex with 12× the most common cytotype in Oregon (Thompson 1997). *Rubus armeniacus* and *R. laciniatus* are part of a tetraploid complex; however, Clark et al. (2013) found *R. armeniacus* to be morphologically indistinguishable from *R. anglocandicans* invasions within Oregon. *Rubus anglocandicans* is reported as pentaploid, but this is potentially the result of a low sample size (Thompson 1997). Higher ploidy number in the blackberry members of *Rubus* could partially explain higher rates of daytime gas exchange, but the disconnect between *g*_day_ and *g*_night_ remains perplexing. Additionally, polyploidy has been frequently correlated with invasive character and could influence invasive success through ecological pre-adaptation, increases in gene pool size, or avoidance of mate limitations on fecundity (te Beest et al. 2012). If genetically driven differences in nighttime transpiration are influencing water-use patterns in invasive *Rubus,* then polyploidy represents an intriguing potential factor influencing *g*_night_. Further work in this group utilizing species with similar ploidy levels will be necessary to separate the direct effect of polyploidy from general ecological or phylogenetic trends in *g*_night_ and *E*_night_.

Differences in nighttime transpiration in this group of *Rubus* species co-occurring in the Pacific Northwest appear to have substantial genetic influence in the absence of water or nutrient stress. *Rubus armeniacus* and *R. laciniatus* showed divergent patterns of gas exchange between day and night relative to *R. spectabilis* and *R. parviflorus*, with lower rates of *g*_night_ and *E*_night_ and higher rates of *g*_day_ and *E*_day_. Phylogenetic patterns of relatedness were evident in all measures of gas exchange and explained a substantial amount of variation in transpiration and conductance at night. Additional examination of diel patterns of water use in other congeneric systems of co-occurring species could provide further insight into divergent patterns of day and nighttime water use. Furthermore, utilizing systems of closely related species with divergent ecological behavior may help determine the potential mechanism (or lack thereof) driving genetic differences in nighttime water use.

## Supporting Information

**Table S1.** Leaf-level anatomical and physiological data for five species of the genus *Rubus* grown in a greenhouse common garden.
