# Supplementary material for: Phylogenetic and ecological patterns in nighttime transpiration among five members of the genus Rubus co-occurring in western Oregon
Source: Ecol Evol. 2015 Aug 6;5(17):3557–69. doi: 10.1002/ece3.1608 (PMC4567861; doi:10.1002/ece3.1608)
Supplement: Supplementary file 1 — Table S1. Leaf-level anatomical and physiological data for five species of the genus Rubus grown in a greenhouse common garden. [file ece30005-3557-sd1.docx]

| **Date** | **Parameter** | **Species** | | | | | **F , p** |
| --- | --- | --- | --- | --- | --- | --- | --- |
|  |  | *R. parviflorus* | *R. spectabilis* | *R. ursinus* | *R. laciniatus* | *R. armeniacus* |  |
| April 2011 | Photo | **11.2424**  ±1.0475 | **12.4661**  ±1.0475 | **15.7204**  ±1.1429 | **18.5000**  ±1.0478 | **20.4928**  ±1.0475 | 19.08, <0.001 |
|  | g_day_ | **0.5682**  ±0.0896 | **0.3200**  ±0.0896 | **0.4109**  ±0.0935 | **0.6595**  ±0.0896 | **0.8400**  ±0.0896 | 8.09, <0.001 |
|  | E_day_ | **7.3057**  ±0.7689 | **4.5145**  ± 0.7689 | **5.5106**  ± 0.8551 | **8.3455**  ± 0.7689 | **9.5613**  ± 0.7689 | 7.56, <0.001 |
|  | g_night_ | **0.0558**  -0.0126,+0.0163 | **0.0450**  -0.0102,+0.0131 | **0.0076**  -0.0018,+0.0023 | **0.0144**  -0.0033,+0.0042 | **0.0159**  -0.0036,+0.0047 | 20.01, <0.001 |
|  | E_night_ | **0.7980**  -0.1798,+0.2320 | **0.6383**  -0.1438,+0.1856 | **0.1285**  -0.0290,+0.0375 | **0.2437**  -0.0549,+0.0709 | **0.2569**  -0.0579,+0.0747 | 19.92, <0.001 |
| July 2011 | Photo | **9.8334**  ±1.5631 | **14.8345**  ±1.5857 | **16.2620** ±1.6815 | **20.6641** ±1.5631 | **23.6678**  ±1.551 | 19.59, <0.001 |
|  | g_day_ | **0.5623**  ±0.1563 | **0.6133**  ±0.1712 | **0.6368**  ±0.1712 | **0.9468**  ±0.1563 | **1.4085**  ±0.1447 | 4.73, 0.006 |
|  | E_day_ | **4.0560**  ±1.0853 | **5.0115**  ±1.1176 | **5.2285**  ±1.1715 | **7.1469**  ±1.0853 | **8.5292**  ±1.0642 | 4.17, 0.011 |
|  | g_night_ | **0.0275**  -0.0034,+0.0039 | **0.0437**  -0.0054,+0.0061 | **0.0209**  -0.0028,+0.0401 | **0.0106**  -0.0015,+0.0017 | **0.0119**  -0.0015,+0.0017 | 19.06, <0.001 |
|  | E_night_ | **0.3762**  -0.0411,+0.0462 | **0.5613**  -0.0614,+0.0689 | **0.3043**  -0.0354,+0.0401 | **0.1656**  -0.0207,+0.0237 | **0.1801**  -0.0198,+0.0221 | 18.39, <0.001 |
|  | Ψ_md_ | **-1.38**  ±0.08 | **-1.41**  ±0.10 | **-1.35**  ±0.09 | **-1.30**  ±0.07 | **-1.16**  ±0.08 | 1.36, 0.277 |
|  | Ψ_pd_ | **-0.63**  -0.08,+0.09 | **-0.57**  -0.08,+0.09 | **-0.55**  -0.08,+0.09 | **-0.38**  -0.05,+0.06 | **-0.27**  -0.03,+0.04 | 6.83, <0.001 |
|  | g_min_ | **0.0056**  ±0.0014 | **0.0075**  ±0.0014 | **0.0055**  ±0.0014 | **0.0031**  ±0.0014 | **0.0053**  ±0.0014 | 1.24, 0.3377 |
| Nov 2011 | SLA | **34.83**  ±1.71 | **29.08**  ±1.71 | **31.46**  ±1.71 | **21.35**  ±1.71 | **31.41**  ±1.71 | 8.79, <0.001 |
| May - June 2013 | Photo | **6.9150**  -0.9668,+1.1239 | **8.9863**  -1.1442,+1.3111 | **12.1436**  -2.0126,+2.4124 |  | **21.1746**  -2.7423,+3.1503 | 21.07, <0.001 |
|  | g_day_ | **0.2161**  ±0.0777 | **0.1901**  ±0.0673 | **0.3151**  ±0.0812 |  | **0.7437**  ±0.0702 | 17.96, <0.001 |
|  | E_day_ | **4.2514**  ±0.8538 | **3.5642**  ±0.7987 | **6.0123**  ±1.1295 |  | **12.0458**  ±0.7987 | 14.35, <0.001 |
|  | g_night_ | **0.0154**  -0.0041,+0.0056 | **0.0171**  -0.0043,+0.0058 | **0.0098**  -0.0033,+0.0049 |  | **0.0034**  -0.0009,+0.0011 | 5.21, 0.007 |
|  | E_night_ | **0.2328**  -0.0605,+0.0818 | **0.2560**  -0.0629,+0.0833 | **0.1512**  -0.0497,+0.0740 |  | **0.0531**  -0.0131,+0.0173 | 5.08, 0.008 |
|  | Ψ_md_ | **-1.12**  ±0.11 | **-1.06**  ±0.11 | **-1.09**  ±0.14 |  | **-0.95**  ±0.10 | 0.97, 0.4254 |
|  | Ψ_pd_ | **-0.24**  -0.04, +0.05 | **-0.20**  -0.03, +0.04 | **-0.31**  -0.07, +0.09 |  | **-0.17**  -0.03, +0.03 | 0.48, 0.6977 |
|  | Ψ_xylem_ | **0.66**  -0.08, +0.09 | **-0.71**  -0.08, +0.09 | **-0.59**  -0.09, +0.11 |  | **-0.60**  -0.08, +0.09 | 0.57, 0.6391 |
|  | Ψ_soil_ | **-0.16**  ±0.03 | **-0.12**  ±0.03 | **-0.06**  ±0.03 |  | **-0.05**  ±0.02 | 3.84, 0.0237 |
|  | stom_density_ | **237.04**  ±31.69 | **219.26**  ±28.35 | **153.09**  ±36.60 |  | **234.57**  ±25.88 | 2.93, <0.0706 |
|  | stom_size_ | **0.001090**  ±0.000060 | **0.000386**  ±0.000054 | **0.000635**  ±0.000060 |  | **0.000553**  ±0. 0.000054 | 27.17, <0.001 |
|  | stom_length_ | **0.03604**  ±0.00153 | **0.02578**  ±0.00137 | **0.03424**  ±0.00177 |  | **0.03401**  ±0.00125 | 18.18, <0.001 |
|  | stom_width_ | **0.03604**  ±0.00151 | **0.01860**  ±0.00135 | **0.02346**  ±0.00174 |  | **0.02038**  ±0.00123 | 29.42, <0.001 |
|  | swc |  | **1.94** ±0.20 | **2.12** ±0.20 |  | **2.22** ±0.20 | 0.5, 0.624 |
|  | Ψ_o_ |  | **-1.05** ±0.05 | **-0.88** ±0.05 |  | **-1.13** ±0.05 | 5.32, 0.03 |
|  | Ψ_TLP_ |  | **-1.23** ±0.06 | -**1.06** ±0.06 |  | **-1.344** ±0.06 | 6.33, 0.019 |
|  | RWC_TLP_ |  | **0.911** ±0.002 | **0.906** ±0.002 |  | **0.901** ±0.002 | 0.16, 0.855 |
|  | c_ft_ |  | **0.541** ±0.121 | **0.924** ±0.121 |  | **0.562** ±0.121 | 3.19, 0.09 |

**Supplemental Table 1:** Leaf-level anatomical and physiological data for five species of the genus *Rubus* grown in a greenhouse common garden. The species are native and non-native co-competitors in Western Oregon. Data are model generated least squares means ±SE. Asymmetrical SE (-,+) were the result of back-transformation after data was log transformed to meet statistical assumptions. Instantaneous gas-exchange parameters were photosynthesis (Photo, µmol m^-2^ s^-1^), stomatal conductance, (g_day_ and g_night_, mol m^-2^ s^-1^), transpiration (E_day_ and E_night_, mmol m^-2^ s^-1^) and minimum stomatal conductance (g_min_, mol m^-2^ s^-1^). Mid-day (Ψ_md,_ MPa), pre-dawn (Ψ_pd,_ MPa), mid-day xylem (Ψ_xylem,_ MPa) and pre-dawn soil (Ψ_soil,_ MPa) water potentials were measured concurrently with gas exchange. Leaf pressure-volume analysis returned six parameters: saturated water content (swc, g H_2_O), osmotic water potential (Ψ_o_, MPa), water potential at leaf turgor loss point (Ψ_TLP_, MPa), relative water content at turgor loss point (RWC_TLP_, %), bulk modulus of elasticity (σ, MPa^-1^), and area-normalized leaf capacitance (c_ft_, mol m^-2^ MPa^-1^). Anatomical traits measured were specific leaf area (SLA, m^2^ kg^-1^), stomatal density (stom_density_, mm^-2^), stomatal size (stom_size_, mm^-2^), stomatal length (stom_length_, mm) and stomatal width (stom_width_, mm).
